# ACL reconstruction in football players: A nationwide Swedish registry study of anteromedial versus transtibial femoral tunnel techniques

**DOI:** 10.1002/jeo2.70818

**Published:** 2026-06-23

**Authors:** Alexander Sandon

**Affiliations:** ^1^ Department of Molecular Medicine & Surgery, Stockholm Sports Trauma Research Center Karolinska Institute Stockholm Sweden

**Keywords:** ACL reconstruction, anteromedial portal, femoral tunnel technique, football (soccer), transtibial

## Abstract

**Purpose:**

To compare anteromedial (AM) portal versus transtibial (TT) femoral drilling techniques in football players undergoing anterior cruciate ligament reconstruction (ACLR), focusing on return‐to‐competition (RTC), match exposure, patient‐reported outcomes (PROMs) and reinjury risk.

**Methods:**

This nationwide cohort study linked the Swedish Knee Ligament Registry with the Swedish Football Association to capture surgical details, verified football participation, and longitudinal PROMs. All index ACLRs in registered football players from 2017–2023 were included. Outcomes were RTC, match exposure, Knee injury and Osteoarthritis Outcome Score and EuroQol 5 dimension score scores at pre‐op, 1, 2 and 5 years, and revision, contralateral, or any second ACL reconstruction. Analyses used t‐tests and *χ* ^2^ tests, and Cox regression adjusted for age, sex, injured side, and meniscal and cartilage injury.

**Results:**

A total of 7169 football players were included (AM 6,653; TT 516). Group differences at baseline were small but present (e.g., more women and left‐sided injuries in TT; more meniscal repair and cartilage injury in AM). RTC rates were similar (55.2% AM vs. 56.8% TT), as were time to RTC, total matches played, matches after RTC, seasons played, and competitive level before and after injury. PROMs showed modest 1‐year differences favouring AM that were not observed at 2 years. No differences were observed at 5 years, although based on limited TT data. Reinjury rates were comparable (revision 6.7% AM vs. 7.4% TT; contralateral 4.6% vs. 4.8%; any second ACLR 11.0% vs. 11.8%). Adjusted Cox models showed no association between portal technique and revision (hazard ratio [HR] 0.878, 95% confidence interval [CI] 0.63–1.23) or any second ACLR (HR 0.906, 95% CI 0.70–1.18), with similar findings in RTC‐only analyses.

**Conclusion:**

In a nationwide cohort of football players, AM and TT femoral drilling techniques yielded similar real‐world outcomes: comparable reinjury risk, RTC rates and timing, match exposure, competitive trajectory, and PROMs. For football players undergoing primary ACL reconstruction, both AM and TT techniques are acceptable options.

**Level of Evidence:**

Level III, retrospective cohort study.

**Abbreviations**
ACL: anterior cruciate ligament
ACLR: anterior cruciate ligament reconstruction
AM: anteromedial
CI: confidence interval
EQ‐5D: EuroQol 5 dimension score
EQ VAS: EuroQol visual analogue scale
HR: hazard ratio
KOOS: Knee injury and Osteoarthritis Outcome Score
LET: lateral extra‐articular tenodesis
MCID: minimal clinically important difference
PROMs: Patient‐Reported Outcome Measures
RTC: return to competition
SD: standard deviation
SKLR: Swedish Knee Ligament Registry
SvFF: Swedish Football Association
TT: transtibial

## INTRODUCTION

Anterior cruciate ligament reconstruction (ACLR) is frequently performed in competitive football players with the aim of restoring knee stability, enabling return to sport, and reducing the risk of subsequent knee injury. Safe and accurate placement of the femoral tunnel is considered a key determinant of rotational stability and graft function [2, 8]. Over the last decade, two primary techniques for femoral tunnel creation have been used in clinical practice: the anteromedial (AM) portal technique, which allows individualised anatomic tunnel placement, and the transtibial (TT) technique, which traditionally offers more constrained tunnel orientation and has been perceived as less anatomic [4, 31].

Biomechanical studies have suggested that AM drilling may improve rotational control and better replicate the native ACL footprint [6, 14], whereas TT drilling has been associated with more vertical tunnel positioning [4, 33]. These theoretical differences have contributed to the assumption that AM drilling should result in improved functional outcomes, higher return‐to‐sport rates, and fewer revision procedures. However, evidence from clinical cohorts remains inconsistent [10, 25, 28, 30], and very few studies have evaluated these techniques specifically in football players, a distinct high‐risk subgroup due to frequent exposure to pivoting and cutting activities and a well‐documented elevated risk of subsequent ACL injury following reconstruction [36]. Whether the theoretical advantages of AM drilling translate into meaningful clinical differences for football players remains unclear.

To address this gap, nationwide registry data from the Swedish Knee Ligament Registry (SKLR) combined with football participation records from the Swedish Football Association (SvFF) was used to evaluate outcomes in a large cohort of football players undergoing ACLR between 2017 and 2023. This allowed comprehensive assessment of surgical results, functional recovery, return to competition (RTC), and reinjury risk using real‐world data.

The aim of this study was to compare clinical outcomes, return‐to‐football patterns, patient‐reported knee function, and the risk of revision or contralateral ACL injury between AM and TT femoral drilling techniques in football players undergoing primary ACL reconstruction. The null hypothesis tested was that there would be no clinically meaningful differences between AM and TT techniques in return to football, match exposure, patient‐reported outcomes, or the risk of revision or contralateral ACL injury.

## METHODS

### Study design and data sources

This retrospective cohort study included all primary ACL reconstructions performed between 1 January 2017 and 31 December 2023 in football players registered in the SKLR. Surgical data, including operative technique, graft choice, meniscal and cartilage findings, and perioperative details, were extracted from the SKLR in April 2025. Information on football participation, match exposure, and competitive level was obtained from the SvFF in April 2025, allowing linkage of pre‐injury exposure, return‐to‐play status, and post‐operative participation.

Players were classified according to the femoral tunnel drilling technique used during ACL reconstruction: AM portal or TT. Procedures without a clearly specified femoral drilling method, multiligament injuries except for grad 1 and 2 medial collateral ligament (MCL), and prior ipsilateral ACLR were excluded. The final study sample consisted of 7169 players, of whom 6653 underwent AM drilling and 516 TT drilling.

### Variables and outcomes

Baseline demographic variables included age at surgery, sex, body mass index (BMI), injured side, and time from injury to surgery. Intra‐articular findings—meniscal injury and treatment, cartilage injury, and graft type—as well as operation time and postoperative follow‐up length were recorded at the time of surgery and follow‐up in SKLR.

The primary outcomes were:

(1) revision ACL reconstruction of the index knee,
(2) contralateral ACL reconstruction, and
(3) any second ACLR, defined as either revision and/or contralateral ACLR, individuals with both events are counted once.

Dates of both primary and subsequent ACLRs were available, enabling time‐to‐event analysis.

Secondary outcomes included RTC after surgery, time to RTC, number of matches before surgery, post‐operative match exposure, seasons played after RTC, and playing level before and after injury. Patient Reported Outcome Measures (PROMs) were assessed using the Knee Osteoarthritis Outcome Score (KOOS) subscales (Symptoms, Pain, Activity and Daily Living (ADL), Sports, Quality of Life (QoL) and EuroQol 5 dimension score (EQ‐5D) index and EuroQol visual analogue scale (EQ‐VAS) at pre‐operative, 1‐year, 2‐year and 5‐year follow‐up.

### Football participation and level of play

Pre‐ and post‐injury level of play for those with available data was classified according to established definitions used in prior SKLR studies [11, 16]. Return to competition was defined as participation in ≥1 official football match after the index ACLR at any level. RTC was verified in the Swedish FA's nationwide competition administration system capturing official match rosters and results, enabling objective verification of RTC. Time to RTC and level at RTC were calculated among players who returned; denominators therefore vary across these descriptive analyses.

### Statistical analysis

Descriptive statistics were generated for all baseline variables, football exposure measures, and PROMs. Continuous variables were summarised as means with standard deviation, categorical variables as counts and percentages.

Group differences between AM and TT techniques were assessed using independent‐samples t‐tests for continuous variables. Levene's test was used to evaluate equality of variances, and when unequal, the Welch t‐test (equal variances not assumed) was used. This applied to all PROMs, surgical time, follow‐up duration, football exposure metrics, and other continuous measures.

Categorical variables including sex, injured side, meniscal pathology, cartilage injury, meniscal treatment, graft type, RTC, revision ACLR, contralateral ACLR and any second ACLR were compared using Pearson's chi‐square test. Fisher's exact test was used when expected cell counts fell below 5.

Time‐to‐event analyses were conducted using Cox proportional hazards regression. Separate models were performed for (1) revision ACLR and (2) any second ACLR. Each model included AM versus TT as the exposure and adjusted for age, sex, injured side, meniscal injury and cartilage injury, reflecting clinically relevant covariates and observed group imbalances. Although additional baseline differences were observed, inclusion of multiple correlated surgical variables would risk multicollinearity and reduce model interpretability. Accordingly, adjustment was limited to key a priori confounders relevant to the study objective. Hazard ratios (HRs) with 95% confidence intervals (CIs) were reported. Because return to competition introduces differential exposure to subsequent ACL injury, Cox models were performed for the entire cohort and separately within the RTC = Yes subgroup.

## RESULTS

A total of 7169 football players undergoing primary ACL reconstruction were included. Of these, 6653 (92.8%) were operated using an AM portal technique and 516 (7.2%) using a TT technique. Baseline characteristics were largely comparable between groups (Table 1). Age at surgery (22.4 ± 6.6 vs. 22.4 ± 7.1 years) and BMI (24.1 ± 3.6 vs. 23.9 ± 4.4 kg/m^2^) were similar between AM and TT, respectively. Several small but statistically significant differences were observed. A greater proportion of females underwent TT reconstruction (40.3%) compared with AM (34.5%) (*p* = 0.007). Left‐sided injuries were slightly more common in TT (50.6%) than AM (45.2%) (*p* = 0.018). Cartilage injury was more frequent in the AM group (20.1%) than in the TT group (9.1%) (*p* < 0.001). The AM technique was associated with higher frequencies of medial and lateral meniscal repair (both *p* < 0.001). AM reconstructions required longer operative time (71.5 ± 26.8 vs. 61.2 ± 20.3 minutes; *p* < 0.001). Graft choice differed by technique: hamstring 86.4% (AM) versus 96.5% (TT), patellar tendon 8.3% versus 3.5% and quadriceps 5.3% versus 0.0% (*p* < 0.001).

**Table 1:** Baseline characteristics of football players undergoing primary ACL reconstruction by femoral drilling technique.

Characteristic		AM portal (*n* = 6653)	Transtibial (*n* = 516)	*p*‐Value
Age at surgery, (m ± SD)	Years	22.42 ± 6.61	22.42 ± 7.09	0.984
Sex, *n* (%)	Female	2292 (34.5%)	208 (40.3%)	0.007
	Male	4361 (65.5%)	308 (59.7%)	
BMI*, (m ± SD)		24.15 ± 3.56	23.94 ± 4.41	0.348
Injured side, *n* (%)	Left	3006 (45.2%)	261 (50.6%)	0.018
	Right			
Time injury → surgery, (m ± SD)	Months	12.15 ± 21.46	7.32 ± 13.58	<0.001
Meniscal injury, *n* (%)	Total	3311 (49.8%)	217 (42.1%)	0.001
	Medial	1638 (24.6%)	105 (20.3%)	0.029
	Lateral	2258 (33.9%)	154 (29.8%)	0.058
Cartilage injury *n* (%)		1340 (20.1%)	47 (9.1%)	<0.001
Meniscal treatment, *n* (%)	Total	2950 (44.3%)	175 (33.9%)	<0.001
Repair (suture), *n* (%)	Medial	828 (12.4%)	33 (6.4%)	<0.001
Repair (suture), *n* (%)	Lateral	726 (10.9%)	32 (6.2%)	0.001
Resection, *n* (%)	Medial	662 (10.0%)	43 (8.3%)	0.235
Resection, *n* (%)	Lateral	1313 (19.7%)	92 (17.8%)	0.293
Graft type, *n* (%)	Hamstring	5747 (86.4%)	498 (96.5%)	<0.001
	Patellar	554 (8.3%)	18 (3.5%)	
	Quadriceps	352 (5.3%)	0 (0%)	
Graft diameter, (m ± SD)	Millimetre	8.7 ± 0.8	8.4 ± 0.8	<0.001
Lateral extra‐articular augmentation (LET)†, *n* (%)		146 (2.2%)	0 (0%)	—
Operative time, (m ± SD)	Minutes	71.54 ± 26.80	61.21 ± 20.28	<0.001
Follow‐up time, (m ± SD)	Years	4.30 ± 1.98	3.99 ± 2.02	0.001

*Note*: Continuous variables are mean ± SD; categorical variables are *n* (%). *p*‐Values from t‐tests (Welch when unequal variances) and Pearson's *χ* ^2^/Fisher's exact tests. *BMI available in subset (AM *n* = 3,177; TT *n* = 287). †LET recorded from mid‐2018; low and asymmetric use.

Abbreviations: ACL, anterior cruciate ligament; AM, anteromedial; BMI, body mass index; SD, standard deviation.

### Football‐related outcomes

Return to play (RTC) rates were similar between groups: 55.2% for AM and 56.8% for TT (*p* = 0.48). Time to RTC also did not differ (501 ± 317 vs. 475 ± 310 days; *p* = 0.18). Football participation, including total games played after surgery, matches before surgery, post‐RTC games and seasons played after RTC were all not statistically different between AM and TT (Table 2). Pre‐injury playing level distributions differed slightly between groups (*χ* ^2^ = 15.65, *p* = 0.01), although the absolute differences were small. Among athletes who returned to football, level change distributions (Higher/Same/Lower) were similar between techniques (*χ* ^2^(2) = 0.914, *p* = 0.633).

**Table 2:** Return‐to‐football and football participation outcomes by femoral drilling technique.

Outcome		AM portal (*n* = 6653)	Transtibial (*n* = 516)	*p*‐Value
Return to football, *n* (%)		3671 (55.2%)	293 (56.8%)	0.48
Time to RTC, m ± SD^a^	Days	501 ± 317	475 ± 310	0.18
Matches, m ± SD	Before surgery	99 ± 78	93 ± 79	0.08
Matches, m ± SD^a^	After surgery	34 ± 37	31 ± 36	0.27
Seasons played after RTC, m ± SD^a^		2.85 ± 1.76	2.74 ± 1.71	0.27
Pre‐injury level, *n* (%)	Top	112 (1.7%)	7 (1.4%)	0.01
	High	956 (14.6%)	95 (18.7%)	
	Middle	1473 (22.5%)	111 (21.8%)	
	Low	2028 (30.9%)	125 (24.6%)	
	Recreational	336 (5.1%)	22 (4.3%)	
	Youth	1653 (25.2%)	149 (29.2%)	
Post‐injury level, *n* (%)	Top	87 (1.3%)	5 (1.0%)	0.35
	High	673 (10.1%)	58 (11.2%)	
	Middle	950 (14.3%)	74 (14.3%)	
	Low	1189 (17.9%)	82 (15.9%)	
	Recreational	326 (4.9%)	27 (5.2%)	
	Youth	443 (6.7%)	47 (9.1%)	
Level change after RTC, *n* (%)	Higher	891 (25.0%)	64 (22.5%)	0.63
	Same	1931 (54.2%)	160 (56.1%)	
	Lower	743 (20.8%)	61 (21.4%)	

*Note*: Continuous variables are mean ± SD; categorical variables are *n* (%). *p*‐Values from t‐tests and *χ* ^2^ tests.

Abbreviations: AM, anteromedial; RTC, return to competition; SD, standard deviation.

^a^ RTC‐only metrics among those who returned (AM *n* = 3,671; TT *n* = 293). Pre‐injury playing‐level percentages use valid cases only (AM *n* = 6,558; TT *n* = 509).

### Patient‐Reported Outcomes (PROMs)

Preoperative KOOS and EQ‐5D scores were equivalent across groups, except for a small difference in KOOS QoL (39.3 vs. 36.8, *p* = 0.044), favouring AM. At 1 year, several KOOS domains favoured AM with small but statistically significant differences: KOOS Symptoms: +4.4 points (*p* = 0.001), KOOS Pain: +3.9 points (*p* = 0.001) and KOOS ADL: +2.6 points (*p* = 0.003). KOOS Sports, KOOS QoL and EQ‐5D/VAS showed no significant differences. By 2 years, most differences had resolved. Only KOOS Symptoms remained statistically different (+ 3.9; *p* = 0.019). All other domains showed no significant differences. At 5‐year follow‐up, no significant differences among available responders were observed between AM and TT in any KOOS or EQ‐5D domain (Table 3).

**Table 3:** Patient‐Reported Outcome Measures (PROMs) by femoral drilling technique (means ± SD and *N*).

PROM	Timepoint	AM portal (mean ± SD, *n*)	Transtibial (mean ± SD, *n*)	*p*‐Value
KOOS symptoms	Pre‐op	70.36 ± 19.57 (*n* = 4349)	68.89 ± 17.79 (*n* = 353)	0.171
	1 year	78.25 ± 16.86 (*n* = 2746)	73.90 ± 19.43 (*n* = 194)	0.001
	2 years	78.50 ± 17.25 (*n* = 1905)	74.57 ± 18.42 (*n* = 132)	0.012
	5 years	79.59 ± 17.85 (*n* = 637)	76.79 ± 21.63 (*n* = 42)	0.332
KOOS Pain	Pre‐op	75.76 ± 18.05 (*n* = 4352)	75.37 ± 17.57 (*n* = 353)	0.694
	1 year	86.52 ± 14.16 (*n* = 2747)	82.62 ± 19.20 (*n* = 194)	0.001
	2 years	86.29 ± 15.01 (*n* = 1905)	84.69 ± 15.38 (*n* = 131)	0.238
	5 years	85.93 ± 16.06 (*n* = 637)	82.47 ± 19.92 (*n* = 42)	0.184
KOOS ADL	Pre‐op	84.25 ± 17.04 (*n* = 4348)	84.36 ± 17.20 (*n* = 353)	0.911
	1 year	93.33 ± 11.40 (*n* = 2747)	90.74 ± 16.52 (*n* = 194)	0.003
	2 years	92.88 ± 12.51 (*n* = 1904)	90.79 ± 14.68 (*n* = 131)	0.068
	5 years	92.05 ± 13.62 (*n* = 637)	91.00 ± 13.51 (*n* = 42)	0.628
KOOS Sports	Pre‐op	45.39 ± 29.00 (*n* = 4342)	44.58 ± 27.68 (*n* = 353)	0.611
	1 year	71.05 ± 23.71 (*n* = 2746)	67.10 ± 28.05 (*n* = 193)	0.027
	2 years	70.05 ± 25.81 (*n* = 1903)	66.49 ± 26.37 (*n* = 131)	0.128
	5 years	70.17 ± 27.17 (*n* = 637)	65.60 ± 29.43 (*n* = 42)	0.293
KOOS QoL	Pre‐op	39.33 ± 22.53 (*n* = 4347)	36.84 ± 19.28 (*n* = 353)	0.044
	1 year	61.30 ± 22.50 (*n* = 2744)	59.41 ± 25.02 (*n* = 194)	0.261
	2 years	61.95 ± 23.95 (*n* = 1902)	60.73 ± 23.63 (*n* = 131)	0.575
	5 years	63.53 ± 25.86 (*n* = 637)	56.25 ± 27.50 (*n* = 42)	0.079
EQ‐5D Index	Pre‐op	0.673 ± 0.243 (*n* = 3780)	0.680 ± 0.237 (*n* = 332)	0.634
	1 year	0.814 ± 0.191 (*n* = 2582)	0.779 ± 0.240 (*n* = 187)	0.017
	2 years	0.816 ± 0.203 (*n* = 1883)	0.782 ± 0.235 (*n* = 131)	0.071
	5 years	0.813 ± 0.220 (*n* = 692)	0.756 ± 0.232 (*n* = 40)	0.118
EQ‐VAS	Pre‐op	60.35 ± 22.77 (*n* = 3570)	60.55 ± 22.49 (*n* = 322)	0.876
	1 year	73.79 ± 19.25 (*n* = 2580)	70.09 ± 23.30 (*n* = 188)	0.012
	2 years	73.3 ± 20.52 (*n* = 1872)	71.8 ± 19.58 (*n* = 129)	0.419
	5 years	71.99 ± 20.67 (*n* = 689)	66.43 ± 22.45 (*n* = 40)	0.100

*Note*: Values are mean ± SD with the number of respondents shown for each timepoint. Analyses use available cases. Statistical tests: t‐test.

Abbreviations: EQ‐5D Index, EuroQol 5 dimension score Index; EQ‐VAS, EuroQol visual analogue scale; KOOS‐ADL, Knee Osteoarthritis Outcome Score‐Activity and Daily Living; KOOS QoL, Knee Osteoarthritis Outcome Score‐Activity and Quality of Life; SD, standard deviation.

### Subsequent ACLR

Across the full cohort, rates of additional ACL surgery were similar between techniques. Revision ACL reconstruction occurred in 444 (6.7%) in the AM group and 38 (7.4%) in the TT group (*p* = 0.546). Contralateral ACL reconstruction occurred in 4.6% (AM) versus 4.8% (TT) (*p* = 0.81). The proportion undergoing any second ACLR (revision or contralateral) was likewise comparable (11.0% AM vs. 11.8% TT; *p* = 0.582). None of the crude between‐group differences were statistically significant (Table 4).

**Table 4:** Reinjury outcomes: Crude rates and adjusted Cox regression results.

Outcome	AM portal (*n* = 6653)	Transtibial (*n* = 516)	*p*‐Value (*χ* ^2^)
Revision ACLR, *n* (%)	444 (6.7%)	38 (7.4%)	0.546
Contralateral ACLR, *n* (%)	307 (4.6%)	25 (4.8%)	0.810
Any second ACLR, *n* (%)	734 (11.0%)	61 (11.8%)	0.582

Adjusted Cox proportional hazards models			
Variable	Hazard ratio (HR)	95% CI	*p*‐Value
Revision ACLR (index knee)	—	—	—
AM vs. TT	0.878	0.63–1.23	0.445
Age (per year)	0.90	0.88–0.92	<0.001
Sex (female)	0.88	0.73–1.07	0.204
Injured side (left)	0.85	0.71–1.02	0.085
Meniscal injury	1.22	1.01–1.47	0.035
Cartilage injury	1.13	0.87–1.46	0.363
Variable	Hazard ratio (HR)	95% CI	*p*‐Value
Any second ACLR (revision + contralateral)	—	—	—
AM vs. TT	0.906	0.70–1.18	0.460
Age (per year)	0.91	0.90–0.93	<0.001
Sex (female)	1.03	0.89–1.19	0.721
Injured side (left)	0.95	0.82–1.09	0.473
Meniscal injury	1.12	0.97–1.29	0.123
Cartilage injury	1.14	0.93–1.38	0.207
Outcome	HR (AM vs. TT)	95% CI	*p*‐Value
Revision ACLR (RTC subgroup)	1.116	0.72–1.72	0.62
Any second ACLR (RTC subgroup)	1.174	0.85–1.62	0.34

*Note*: Crude reinjury outcomes are presented as *n* (%). Adjusted Cox models report hazard ratios (HR) with 95% confidence intervals (CI) and *p*‐values. Models adjust for age, sex, injured side, meniscal injury, and cartilage injury. “Any second ACLR” includes either revision of the index knee or contralateral ACL reconstruction. RTC‐only models include athletes with postoperative exposure after return to football.

Abbreviations: ACLR, anterior cruciate ligament reconstruction; AM, anteromedial; RTC, return to competition; TT, transtibial.

Cox proportional hazards analyses showed the same pattern. In the full cohort, femoral drilling technique was not associated with the hazard of revision ACL reconstruction (HR = 0.878, 95% CI 0.63–1.23; *p* = 0.445). In analyses restricted to players who returned to football there was likewise no difference (HR = 1.116, 95% CI 0.72–1.72; *p* = 0.62). For any second ACLR, AM and TT again did not differ (HR = 0.906, 95% CI 0.70–1.18; *p* = 0.46), with consistent results in the RTC subgroup (HR = 1.174; *p* = 0.34).

Across all adjusted models, older age showed a consistent protective association (HR ≈ 0.90 per year; *p* < 0.001), while sex, injured side, meniscal injury, and cartilage injury were not consistently associated with the likelihood of revision or any second ACL reconstruction.

## DISCUSSION

The principal finding of this study is that femoral tunnel drilling technique, anteromedial portal versus transtibial, was not associated with clinically meaningful differences in reinjury risk, return‐to‐football, or medium‐term patient‐reported outcomes in Swedish football players undergoing primary ACL reconstruction. These findings suggest that, in contemporary practice, portal technique alone does not appear to be a dominant determinant of outcome in this high‐demand population.

### Return to football and post return participation

The observed RTC rate of approximately 55%–57% is consistent with previous population‐based studies of Swedish football players after ACL reconstruction [36], including prior work using combined SKLR and SvFF data [11, 16, 37]. The present findings therefore reinforce that RTC rates in football remain constrained by factors beyond surgical technique, such as reinjury fear, competing life priorities, persistent symptoms, and the physical demands of the sport [12, 13, 36].

Importantly, neither RTC rate nor time to RTC differed between AM and TT techniques, nor did post‐RTC match exposure, number of seasons played, or competitive level achieved. These data extend previous RTC literature by demonstrating that portal technique does not modify real‐world football participation [29], even when exposure is quantified objectively rather than inferred from self‐reported activity scales [34].

### Patient‐reported outcomes

Small differences in PROMs favouring the AM technique were observed at 1‐year follow‐up in selected KOOS subscales. However, these differences were below established patient Minimal clinically important difference (MCID) thresholds and therefore unlikely to be clinically meaningful [15, 26, 38]. Moreover, these early differences diminished at 2 years and were no longer present at 5‐year follow‐up, where PROMs were fully convergent between techniques. This is in contrast with results from the MARS group showing favourable KOOS scores for the TT group after 6 years [39]. Others have like in the present study find stabilisation of the scores after 2 years and a ceiling effect up to 10 years [27, 36]. From a clinical perspective, the absence of long‐term PROM differences reinforces that portal technique choice does not influence patient‐perceived knee function or quality of life in the medium to long term. PROM response rates declined over time, particularly at 5 years, which is a recognised limitation of registry‐based studies [5]. However, this degree of attrition is comparable to other SKLR‐based publications and to large national ACL registries internationally [3, 7, 21]. Furthermore, PROMs were a secondary outcome in this study, and dropout was similar between groups, reducing the likelihood of differential attrition bias affecting between‐technique comparisons.

### Reinjury risk and exposure‐adjusted interpretation

Neither crude proportions nor multivariable time‐to‐event analyses demonstrated differences in revision ACL reconstruction, contralateral ACL reconstruction, or any second ACLR between AM and TT techniques. This remained true when analyses were restricted to players who returned to football, thereby accounting for differential exposure to reinjury risk. These findings are notable given the high rotational demands of football and the theoretical biomechanical advantages attributed to AM drilling. The lack of observed differences suggests that any potential biomechanical distinctions are insufficient to translate into measurable differences in reinjury risk under real‐world conditions, where exposure, age and behavioural factors play dominant roles.

Although graft choice was not specifically examined in this study, it is noteworthy that most reconstructions were performed using hamstring autografts (86.4% AM, 96.5% TT). Despite this, revision rates were low in this large cohort of football players with objectively verified RTC. While no causal inferences can be made, these findings suggest that concerns regarding higher failure risk with hamstring grafts may be less prevalent than previously perceived.

### AM versus TT: Evolution of technique rather than technique superiority

Historically, the shift from TT to AM drilling was driven by biomechanical and cadaveric studies demonstrating more anatomic femoral tunnel placement and improved rotational control with AM techniques. However, the present findings highlight that technique labels may no longer reliably reflect tunnel position or functional outcome [9, 18].

Several explanations likely contribute:

First, modern TT techniques have evolved. Modified TT approaches allow surgeons to achieve less vertical, more anatomic femoral tunnels than those produced by traditional TT drilling [1, 35, 41]. As a result, the historical biomechanical disadvantages of TT may no longer apply uniformly in contemporary practice [17, 42].

Second, AM drilling does not guarantee anatomic tunnel placement [23, 32]. Femoral tunnel position using the AM portal is highly operator‐dependent and influenced by knee flexion angle, portal placement, visualisation, and surgeon experience [20, 24]. Variability in AM technique may therefore reduce the expected consistency of anatomic placement.

Third, the importance of striving for an anatomic placement is desired regardless of technique in moderns ACLR and once a threshold of acceptable tunnel position is reached [17, 19, 40], further refinements in femoral orientation may yield diminishing clinical returns. In this context, patient factors, biological healing, neuromuscular recovery, and exposure to risk activity likely outweigh small technical differences in tunnel geometry [11].

Together, these considerations suggest that the observed similarity between AM and TT reflects convergence of real‐world surgical practice, rather than failure of biomechanical theory per se. For football players undergoing ACL reconstruction, these data support a technique‐neutral approach to femoral tunnel drilling. Surgeons can reasonably select AM or TT techniques based on familiarity, safety, and the overall surgical strategy, without expectation of differences in return to football, reinjury risk, or medium‐term knee‐specific quality of life. From an athlete counselling perspective, these findings allow clinicians to shift emphasis away from portal technique and toward factors with greater demonstrated impact, including rehabilitation quality, readiness to return, exposure management and long‐term knee health [11, 22].

### Limitations

This study has several limitations that should be considered when interpreting the findings. First, the observational design introduces potential selection bias. Portal technique was not randomly assigned, and although baseline characteristics were largely similar, meaningful differences existed TT was used in a smaller proportion of cases, with a higher percentage of female players and slightly more left‐sided injuries, whereas AM procedures demonstrated higher rates of meniscal repair and cartilage injury and required longer operative time. These imbalances suggest that surgeons may preferentially choose AM or TT. Second, while the study benefits from detailed surgical and sport‐exposure data, it lacks radiographic or intraoperative measurements of tunnel position. As such, we cannot determine whether the AM and TT groups achieved distinct anatomic tunnel orientations as assumed, especially given advances in TT techniques and variability in AM drilling. Objective assessment of tunnel placement would be necessary to more directly link tunnel geometry with reinjury risk. Third, although the dataset includes granular football exposure, an important strength, it does not capture several influential post‐operative factors: rehabilitation quality, neuromuscular recovery, strength symmetry, psychological readiness, training load, or compliance with return‐to‐sport criteria. These variables substantially influence reinjury risk and return‐to‐play behaviour and may overshadow minor biomechanical differences introduced by surgical technique. Fourth, PROM follow‐up attrition increased with time, particularly at 5 years. While loss to follow‐up was similar between groups, the reduced sample limits sensitivity to detect small but potentially meaningful medium‐term differences, and the results may be more reflective of players who remain engaged with the healthcare system. Fifth, the study does not differentiate among sub‐variants of the TT technique (e.g., modified or independent TT), nor does it account for surgeon volume or experience. Given the small TT sample and evolving technical approaches during the study period, TT procedures may represent a heterogeneous group ranging from traditional vertical tunnels to more refined anatomic placements. Sixth, although the study focuses exclusively on football players it does not track training exposure, match minutes, or position‐specific demands, all of which might influence reinjury risk. Total matches and seasons provide useful load estimates but cannot fully characterise exposure intensity or contextual match demands. Finally, while national registry data provide high external validity, they rely on clinician‐reported fields. Variability in documentation of intra‐articular pathology, surgical details, and postoperative findings introduces the possibility of misclassification, especially regarding meniscal and cartilage injuries. However, SKLR reporting has historically demonstrated high overall completeness, and the large sample size mitigates random error.

## CONCLUSION

In a nationwide cohort of football players, AM and TT femoral drilling techniques yielded equivalent real‐world outcomes: comparable reinjury risk, RTC rates and timing, match exposure, competitive trajectory, and PROMs. For football players undergoing primary ACL reconstruction, both AM and TT techniques are acceptable options.

## AUTHOR CONTRIBUTIONS

A.S. conceived the study, performed all analyses, interpreted the data, and wrote the manuscript.

## FUNDING INFORMATION

The author has no funding to report.

## CONFLICT OF INTEREST STATEMENT

The author declares no conflicts of interest.

## ETHICS STATEMENT

Approved by the Swedish Ethical Review Authority (Dnr 2024‐06108‐01).

## Data Availability

Data were obtained from the Swedish Knee Ligament Registry (SKLR) and the Swedish Football Association (SvFF). Access requires approval from the Swedish Ethical Review Authority and registry owners.
